# D-Tagatose Feeding Reduces the Risk of Sugar-Induced Exacerbation of Myocardial I/R Injury When Compared to Its Isomer Fructose

**DOI:** 10.3389/fmolb.2021.650962

**Published:** 2021-04-13

**Authors:** Mariaconcetta Durante, Silvia Sgambellone, Laura Lucarini, Paola Failli, Annunziatina Laurino, Debora Collotta, Gustavo Provensi, Emanuela Masini, Massimo Collino

**Affiliations:** ^1^Department of Neuroscience, Psychology, Drug Research and Child Health (NEUROFARBA), Section of Pharmacology and Toxicology, University of Florence, Florence, Italy; ^2^Department of Drug Science and Technology, University of Turin, Turin, Italy

**Keywords:** fructose, myocardial ischemia, inflammation, oxidative stress, D-tagatose

## Abstract

It is known that fructose may contribute to myocardial vulnerability to ischemia/reperfusion (I/R) injury. D-tagatose is a fructose isomer with less caloric value and used as low-calorie sweetener. Here we compared the metabolic impact of fructose or D-tagatose enriched diets on potential exacerbation of myocardial I/R injury. Wistar rats were randomizedly allocated in the experimental groups and fed with one of the following diets: control (CTRL), 30% fructose-enriched (FRU 30%) or 30% D-tagatose-enriched (TAG 30%). After 24 weeks of dietary manipulation, rats underwent myocardial injury caused by 30 min ligature of the left anterior descending (LAD) coronary artery followed by 24 h′ reperfusion. Fructose consumption resulted in body weight increase (49%) as well as altered glucose, insulin and lipid profiles. These effects were associated with increased I/R-induced myocardial damage, oxidative stress (36.5%) and inflammation marker expression. TAG 30%-fed rats showed lower oxidative stress (21%) and inflammation in comparison with FRU-fed rats. Besides, TAG diet significantly reduced plasmatic inflammatory cytokines and GDF8 expression (50%), while increased myocardial endothelial nitric oxide synthase (eNOS) expression (59%). Overall, we demonstrated that D-tagatose represents an interesting sugar alternative when compared to its isomer fructose with reduced deleterious impact not only on the metabolic profile but also on the related heart susceptibility to I/R injury.

## Introduction

The dramatic rise in the prevalence of metabolic disorders has occurred in parallel with an escalation in dietary sugar consumption. Indeed, the consumption of added sweeteners containing fructose has increased dramatically in the last 40 years (Newens and Walton, 2016; Taskinen et al., 2019), and clinical trials as well as experimental studies suggest that high fructose intake is an important causative factor of metabolic derangements associated with an excessive inflammatory response and oxidative stress (Mellor et al., 2010). Accordingly, fructose consumption has been associated to higher prevalence of obesity, type 2 diabetes mellitus (T2DM), and cardiovascular-related complications (Hu and Malik, 2010; Lim et al., 2010; Rizkalla, 2010; DiNicolantonio et al., 2015). Cardiovascular diseases (CVD) are the most prevalent cause of morbidity and mortality in T2DM patients (Henning, 2018), and ischemic heart disease is one of the most common cause of death in T2DM patients. There is substantial evidence that exposure to excessive fructose intake has detrimental effects on multiple cardiometabolic risk factors such as insulin resistance, intrahepatic lipid accumulation and hypertriglyceridemia (Taskinen et al., 2019).

To achieve the best results in preventing nutrition-related CVD maintaining a normal endothelial function, innovative strategies, aimed to replace conventional sugars with new low caloric sweeteners, have gained increasing attention. One of the most promising alternative sugar is D-tagatose, a rare natural ketohexose commonly produced from microbial bioconversion of D-galactose. D-tagatose is an isomer of fructose and its sweetness is equivalent to 90% that of sucrose, with low caloric intake (<1.5 kcal/g, 33% of the calorie of sucrose), minimal glycemic effect (Noronha et al., 2018) and growth promoting effects on beneficial gut bacteria (Roy et al., 2018). Since its classification as a generally recognized safe product (GRAS) by the Food and Drug Administration (FDA) in 2001, it has been used as a nutritional sweetener (Levin, 2002) to control glycemia (Guerrero-Wyss et al., 2018).

Preliminary animal studies showed the ability of D-tagatose to lower blood glucose and lipoprotein levels, generating great interest in the scientific community (Yadav et al., 2018). D-tagatose acts as a “sugar blocker” by preventing lipid formation from carbohydrates without stimulation of pancreatic beta cells for insulin production and secretion (Mooradian, 2019). Besides, it has been shown to increase high-density lipoprotein (HDL) levels (Donner et al., 2010). Results obtained in T2DM patients reported that supplementation with D-tagatose resulted in lower fasting blood glucose and HbA1c, and lower LDL and total cholesterol (TC; Ensor et al., 2015). The molecular mechanisms underlying the beneficial and healthy effects of D-tagatose are not completely understood. Recently, we reported a significant reduction of the metabolic abnormalities in mice chronically fed with D-tagatose when compared to fructose (Collotta et al., 2018). These differences were due, at least in part, to the lower chemical reactivity of D-tagatose when compared to its isomer fructose, resulting in lower local accumulation of advanced glycation end-products (AGEs), a heterogeneous group of compounds with multiple biological effects that can contribute to the development and progression of *ex novo* lipogenesis and inflammation (Cepas et al., 2020). As we previously documented (Mastrocola et al., 2013, 2018; Collotta et al., 2018), slight differences in dietary sugars may significantly affect the evolution of metabolic diseases. Despite the close link between metabolic derangements and development of cardiovascular injuries, so far, no experimental data are available on the potential impact of the reduced susceptibility to metabolic derangements evoked by D-tagatose when compared to fructose on exacerbation of myocardial infarction. Thus, this study aims to extend our investigation from the metabolic to the cardiometabolic context, showing if and how chronic D-tagatose feeding resulted in a reduced risk of exacerbation of the injury evoked by an ischemic insult in comparison to the results obtained with fructose feeding. We also investigated the potential differences of the two fructose isomers in terms of activation of oxidative stress and inflammatory cascades specifically involved in the cardiac dysfunction following myocardial ischemia-reperfusion (I/R) injury.

## Materials and Methods

### Materials

D-tagatose was synthesized by Inalco RSM S.p.a, Research Center (Montale, Pistoia, Italy), while fructose was acquired from Sigma-Aldrich (St. Louis, MO, United States). Animal diets were prepared by Sniff (Sniff Spezialdiäten, GmbH, Soest, Germany) and had the following composition: control diet (CTRL) was prepared 70% calories in carbohydrates (44.2% wheat flour, 14.9% dextrin and 11% sugar), 8.1% fat, 4.1% fiber, and 17.8% protein. Fructose-enriched (FRU 30%) or D-tagatose-enriched (TAG 30%) diets: 70% calories in carbohydrates [25.1% wheat flour, 14.9% dextrin, and 30% sugars (fructose or tagatose, respectively)], 10% fat, and 20% proteins.

### Study Design

**FIGURE 1:**
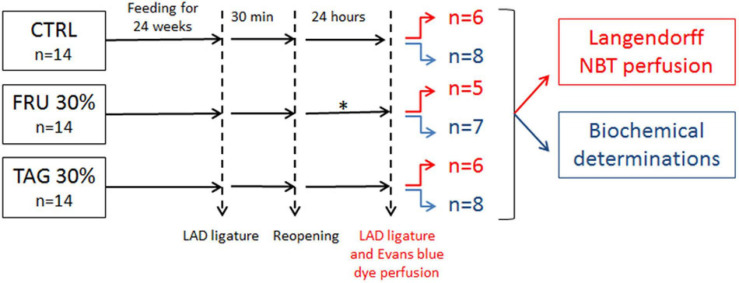
Flowchart of study design. The subgroups of animals represented in red were subjected to left anterior descending (LAD) ligature and nitro-blue tetrazolium (NBT) perfusion. The subgroups of animals subjected to biochemical determinations are indicated in blue. ^∗^2 rats in fructose (FRU 30%) group died after 12 and 15 h of reperfusion.

Male albino Wistar rats (8–10 weeks old) were purchased from Charles River Laboratories (Wilmington, MA, United States). At the University of Florence (Centre for Laboratory Animal Housing and Experimentation), rats were housed in pairs with food and tap water *ad libitum* and standardized conditions of temperature, humidity and light. The experiments have been performed in keeping with the Council Directive of the European Community (2010/63/EU), with the Italian Legislative Decree 26 (13/03/2014), and the protocol has been approved by the animal Ethical and Care Committee of the University of Florence (Florence, Italy) and the Italian Health Ministry (Authorization n 1189/2016-PR). Rats were allocated in three dietary regimen groups for 24 weeks according to the method described by (Collotta et al., 2018): a group fed with a control diet (CTRL *n* = 14), a group fed with 30% fructose diet (FRU 30% group, *n* = 14) and a group fed with 30% D-tagatose diet (TAG 30% group, *n* = 14) (Figure 1).

### Surgical Procedure

A 22-gauge cannula (0.8 mm diameter, Venflon 2; BD Biosciences, Franklin Lakes, NJ, United States) was inserted into the trachea of anaesthetized rats (ketamine, Lobotor, Acme S.r.l., 80 mg kg^–1^ b.w., plus xylazine, Dechra Veterinary Products S.r.l., 10 mg Kg^–1^ b.w., ip) and air ventilation was performed using a Palmer pump (Ugo Basile, Comerio, Italy) at a rate of 40–45 stroke per minute and a volume of 4 ml/stroke. To prevent hypothermia, a water warmed surgical bed (Kent Scientific Corporation, Torrington, CT, United States) was used during surgical procedures. Then, rats underwent thoracotomy at the fifth left intercostal space, the pericardium was opened, and a loose 00-braided silk suture was placed around the left anterior descending (LAD) coronary artery, 1–2 mm below its origin. In order to simplify the removal of the suture at the end of the ischemia, a small silicon ring was inserted in the silk thread below the knot. Then, to minimize heart displacement, the chest was closed by a silk suture, leaving the ends of the coronary suture threads emerging from the surgical wound. Ischemia (30 min) was induced by tightening the threads of the coronary suture, whereas, reperfusion (24 h) was obtained by reopening the chest and cutting the ligature around the coronary artery (Collino et al., 2012). Survival time was recorded in all animals. Two rats (2/14) died after 12 and 15 h of reperfusion in the FRU 30% group.

### Blood and Tissue Sampling

Tissues and tail vein blood were collected at the end of reperfusion. Rats were euthanized and whole blood was collected. Samples were centrifuged (1,200 × *g*, 4°C, 15 min). Plasma and serum were separated and then stored at −80°C for the following analysis. Hearts, liver and kidneys were harvested, divided into aliquots and stored at −80°C for biochemical determinations or fixed in 4% paraformaldehyde for subsequent histopathological analysis. A hearth isolated from a Naïve rat was used as morphological reference.

### Assessment of the Size of the Infarct

After 24 h, the animals were anesthetized again. In a subgroup of animals from each experimental group (8 rats for CTRL and TAG 30% groups, and 7 rats for FRU 30% group), the heart was quickly removed and frozen for successively biochemical determinations; while another subgroup (6 rats for CTRL and TAG 30% groups, and 5 rats for FRU 30% group) was submitted to the re-occlusion of LAD coronary artery and 0.25% Evans blue dye was injected into the coronary system *in vivo* for the measurement of the at risk area (AAR) (Masini et al., 2002). After, the heart was quickly removed from the thorax, a cannula was introduced into the aorta and the hearts were attached to a Langendorff apparatus and perfused with 10 ml of 1% nitro-blue tetrazolium (NBT) dissolved in a modified Tyrode solution, pH 7.4, at a constant pressure of 40 cm of water at 37°C for 20 min. The hearts were detached from the cannula, weighed, and the ventricles cut from the apex to the base into 2 mm slices. A superimposed acetate sheet was used to trace the bound areas of the unstained area on the upside surface of each slice and the encircled area was measured by computer-assisted morphometry. The total volume of the damaged myocardium was determined, as previously described (Masini et al., 2002).

### L-Hydroxyproline Assay

The L-hydroxyproline content indicates collagen deposition, which is utilized to assess tissue damage. Samples from frozen cardiac tissue were previously lyophilized for 48 h and then thoroughly homogenized in distilled water. The samples were gently mixed with 12 M hydrochloric acid and hydrolyzed by autoclaving at 120°C for 40 min. After evaporation the samples were reconstituted in 2 ml of distilled water. Each sample was oxidized adding 1 ml of chloramine-T for 20 min at room temperature. Chloramine T was neutralized with 1 ml of 3.0 M perchloric acid. The samples were mixed with 1 ml of p-dimethylaminobenzaldehyde and incubated at 65°C for 20 min and then cooled at room temperature afterward. The absorbance was measured at 550 nm and the L-Hydroxyproline concentration was measured using a standard curve and expressed as μg/mg of protein (Lucarini et al., 2017). Total protein levels were measured using BCA Protein Assay (Thermo Fisher Scientific, Waltham, MA, United States).

### Histopathological Analysis

The paraffin-embedded tissue sections (6 μm) of heart tissue were stained with AZAN staining method. In order to minimize artifactual differences in the staining process, all sections were stained in a single session. A digital camera connected to a light microscope equipped with a × 40 objective was used to randomly take photomicrographs of the histological slides.

### Plasmatic Metabolic Parameters

Rats were fasted overnight and blood samples were collected from the tail vein to measure the blood glucose level using a Glucocard MX Blood Glucose Meter (A. Menarini Diagnostic, Florence, Italy). Serum concentrations of total cholesterol (TC), high-density lipoprotein cholesterol (HDL), and triglycerides (TG) were enzymatically measured with specific reagent kits (Bayer, Pittsburgh, PA, United States), using an automatic chemistry analyzer (ADVIA 1650; Bayer, Osaka, Japan). Serum low-density lipoprotein cholesterol (LDL-C) concentrations were quantified by the Friedwald equation, subtracting the HDL-C from the TC concentration (Friedewald et al., 1972).

Serum insulin levels were assayed with Rat/Mouse Insulin ELISA kit (Millipore Merck, Darmstardt, Germany); the amount of insulin is measured spectrophotometrically, by interpolation of absorbance from a reference curve with reference standards of known concentrations of rat insulin. The insulin resistance index was calculated using the homeostasis model assessment (HOMA) index with the following formula:

Homa-IR=glucoseconcentration(mg/dL) × insulin(μU/mL)/405(Matthews et al., 1985).

### Cytokines Determination

The quantitative determination of the interleukin-6 (IL-6), interleukin-1β (IL-1β), and tumor necrosis factor (TNF)-α was performed by a bead-based multiplex immunoassay, following the manufacturer protocol (EDM Millipore Corporation, Billerica, MA, United States). Briefly, plasma samples were added to antibody-conjugated beads directed against the cytokines listed above in a 96-well filter plate. After a 30-min incubation, the plate was washed, and biotinylated anti-cytokine antibody solution was added for overnight incubation. Following incubation with streptavidin-conjugated PE each well was analyzed with the Bioplex 200 system. Cytokine quantification was performed using standard curves obtained by using cytokine standards that underwent the same protocol as the plasma samples. Values are indicated as mean ± SD for each group and expressed as pg/mL.

### Malonyldialdehyde Determination

Malonyldialdehyde (MDA), an end-product of lipid peroxidation of cell membrane caused by oxygen derived free radicals, was determined by measurement of the chromogen obtained from the reaction of MDA with 2-thiobarbituric acid. Approximately 100 mg of tissue were homogenized with 1 ml of Tris HCl buffer (50 mmol/L) containing 180 mM KCl and 10 mM EDTA, final pH 7.4, using a tissue homogenizer (Ing. Terzano, Milan, Italy). Then 0.5 ml of 2-thiobarbituric acid (1% w/v) in 50 mM NaOH and 0.5 ml HCl (25% in water) were added to the sample. The mixture was then heated in boiling water for 10 min. The chromogen was extracted with 3 ml of 1-butanol and the organic phase separated by centrifugation and spectrophotometrically quantified at 532 nm wavelength. Values are expressed as nanomoles of thiobarbituric acid-reactive substances (MDA equivalents) per milligram of protein, using a standard curve of 1,1,3,3-tetramethoxypropane (Bani et al., 1998). Total protein concentration was measured using Micro BCA Protein Assay (Thermo Fisher Scientific, Waltham, MA, United States).

### 8-Hydroxy-2′-Deoxyguanosine Determination

DNA isolation from cardiac tissue homogenates was performed as previously described (Collotta et al., 2018). Frozen samples were homogenized in 1 ml of 10 mM PBS, pH 7.4, sonicated on ice for 1 min, added with 1 ml of 10 mM Tris HCl buffer, pH8, containing 10 mM EDTA, 10 mM NaCl, 0.5% SDS, and incubated for 1 h at 37°C with 20 μg/ml RNase 1 (Sigma-Aldrich, St. Louis, MO, United States). The samples were then incubated overnight at 37°C with100 μg/ml proteinase K (Sigma-Aldrich, St. Louis, MO, United States). The DNA extraction was performed with chloroform/isoamyl alcohol (10:2, v/v). The extracted DNA was precipitated from the aqueous phase with ammonium acetate, solubilized in 200 μl of 20 mM acetate buffer, pH 5.3, and denatured at 90°C for 3 min. The extract was supplemented with 10 IU of P1 nuclease (Sigma-Aldrich, St. Louis, MO, United States) in 10 μl, and incubated for 1 h at 37°C with 5 IU of alkaline phosphatase (Sigma-Aldrich, St. Louis, MO, United States) in 0.4 M phosphate buffer, pH 8.8. The samples were filtered with an Amicon Micropure-EZ filter (Amicon, MA, United States). The 8-Hydroxy-2′-Deoxyguanosine (8-OH*d*G) concentration was measured in 50 μl of each sample by using the Highly Sensitive 8-OH*d*G Check ELISA kit (JalCA, Shizuoka, Japan). The results were expressed as ng of 8-OH*d*G/ng of total DNA.

### Western Blot Analysis

A radioimmunoprecipitation assay (RIPA) buffer enriched in protease inhibitors was used for tissue homogenization. Homogenates were centrifuged at 14,000 × *g* for 10 min and total protein levels were measured in the collected supernatants using BCA Protein Assay (Thermo Fisher Scientific, Waltham, MA, United States). Fifty μg of proteins were separated by SDS-PAGE and electro-transferred on PVDF membranes, which were then incubated with the following rabbit monoclonal antibodies: anti-COX-2 and anti- endothelial nitric oxide synthase (eNOS) (1:500, Santa Cruz Biotechnology, Santa Cruz, CA, United States), anti-GDF8 (1:1,000, Biorbyt Ltd., Cambridge, United Kingdom), followed by incubation with appropriated HRP-conjugated secondary antibodies. The loading transfer of equal amounts of proteins was checked by reblotting the membrane with anti-β-actin antibody (1:50,00; Sigma-Aldrich, St. Louis, MO, United States). Bands were visualized by enhanced chemiluminescence (ECL; Thermo Fisher Scientific, Waltham, MA, United States) and quantified by densitometric analysis with the ImageJ software.

### Statistical Analysis

Data are expressed as mean ± SD and statistical analysis (one-way ANOVA followed by Bonferroni multiple comparison test) were performed using GraphPad Prism 6 (GraphPad Software, San Diego, CA, United States), *p* < 0.05 was considered significant.

## Results

### Comparative Analysis of the Impact of Two Sugar-Enriched Diets on Metabolic Parameters

**FIGURE 2:**
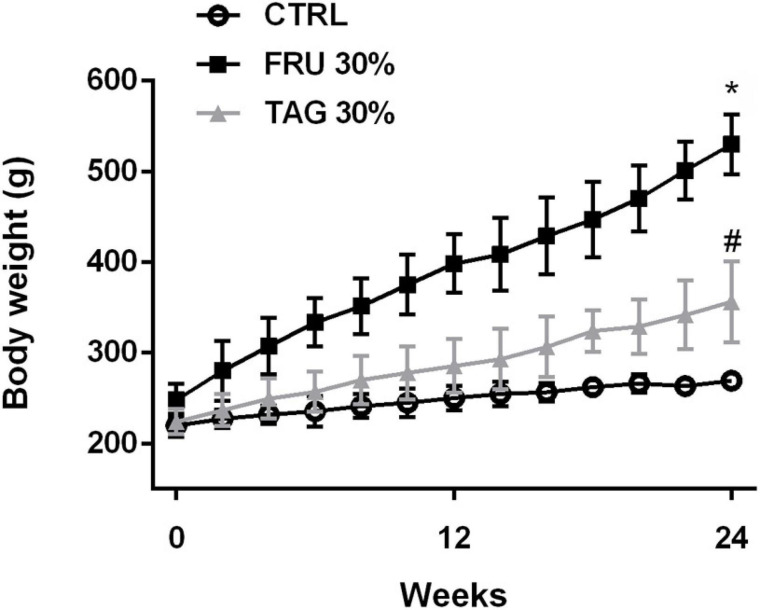
Body weight gain in rats fed with controls (CTRL), D-tagatose (TAG 30%) and fructose (FRU 30%) groups for 24 weeks. Data are means ± SD for 14 animals per group. *p < 0.05 vs. CTRL, #*p* < 0.05 vs. FRU 30%.

Rats chronically fed with FRU 30% diet for 24 weeks gain significantly more weight than CTRL rats (Figure 2). Interestingly, body weight gain recorded in rats exposed to TAG 30% was significantly lower than observed in FRU 30% diet-fed rats. Consumption of the fructose-enriched diet resulted in increased glycaemia, insulin level, serum TC, triglycerides and LDL-cholesterol, associated with a significant decrease in HDL-cholesterol levels. Abdominal fat was increased in FRU 30% diet fed rats. Similarly, rats exposed to FRU 30% diet had significantly elevated insulin resistance index compared with either the CTRL or TAG 30% group. On the contrary, the level of the glucose, the lipid profiles recorded in the blood, the abdominal fats, the insulin level and the insulin resistance index of rats chronically exposed to D-tagatose were not significantly different from those recorded in the control group (*p* > 0.05) (Table 1).

**TABLE 1:** Effects of fructose (FRU 30%) and tagatose (TAG 30%) enriched diets in comparison to a standard diet (CTRL) on glucose and lipid metabolism.

	**CTRL (*n* = 14)**	**FRU 30% (*n* = 12)**	**TAG 30% (*n* = 14)**
Glucose (mg/dL)	101.3 ± 4.32	286.2 ± 8.8*	114.5 ± 3.1^#^
Total Cholesterol (mg/dL)	53.36 ± 2.85	75.02 ± 3.51*	58.1 ± 4.8^#^
HDL (mg/dL)	33.91 ± 2.7	15.9 ± 3.1*	31.8 ± 2.4^#^
LDL (mg/dL)	17.02 ± 2.1	46.35 ± 2.9*	24.1 ± 3.7^#^
Triglyceride (mg/dL)	61.6 ± 1.9	195.8 ± 4.9*	107.86 ± 8.4^#^
Abdominal fat/body weight (%)	6.2 ± 1.6	11.3 ± 2.0*	8.6 ± 1.2^#^
Insulin (μU/mL)	3.63 ± 0.92	7.59 ± 1.81*	3.9 ± 0.84^#^
Insulin Resistance Index	0.91 ± 0.26	5.36 ± 1.4*	1.10 ± 0.26^#^

*p < 0.05 vs. CTRL; #*p* < 0.05 vs. FRU 30%.

### Effect of D-Tagatose on Infarct Size and Collagen Tissue Levels in Rats Exposed to Myocardial Ischemic Injury

**FIGURE 3:**
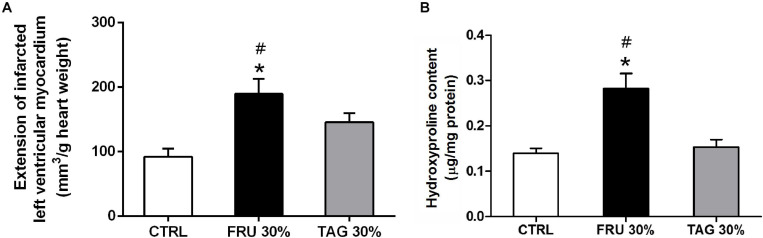
Impact of chronic sugar exposure on myocardial damage. **(A)** Extension of myocardial infarct size. The extension of infarcted left ventricular myocardium was evaluated on hearts stained with nitro-blue tetrazolium (NBT) after left anterior descending (LAD) occlusion. Data are means ± SD for 6 (CTRL and TAG 30% groups) and 5 (FRU 30% group) animals per group. **(B)** Hydroxyproline content in heart tissue homogenates. Data are means ± SD for four samples per group; *p < 0.05 vs. CTRL; #*p* < 0.05 vs. TAG 30%.

Chronic D-tagatose consumption improved the survival at the end of 24-h reperfusion. In fact, no animal died in this group as well as in the control group, while 2 rats (2/14) died after 12 and 15 h of reperfusion in the FRU 30% group. As shown by computer-assisted morphometric analysis (Figure 3A), the heart of rats fed with FRU 30% showed an infarct volume significantly higher than that recorded in the heart of CTRL animals. Although the extension of myocardial damage in TAG 30%-fed rats was slight greater than observed in the CTRL group it did not reached statistical significance. Most importantly, it was significantly lower in comparison with FRU 30%-fed rats.

The levels of hydroxyproline content were significantly increased in heart tissue from FRU 30% rats when compared to those recorded in CTRL group. In contrast, chronic exposure to the TAG 30% diet did not significantly increase (*p* > 0.05) hydroxyproline level in comparison to CTRL diet (Figure 3B).

**FIGURE 4:**
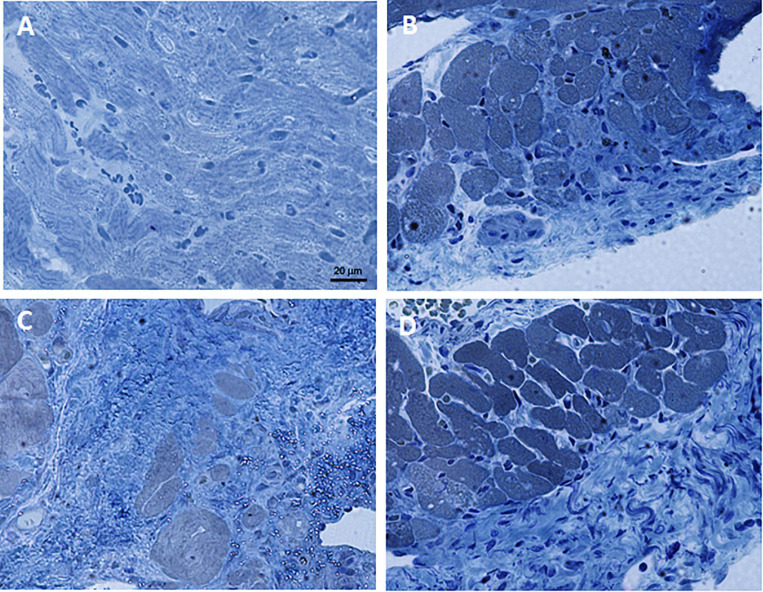
Representative images of AZAN-stained histological sections of heart tissue samples. **(A)** Naïve. **(B)** Ischemia/reperfusion CTRL group. **(C)** Ischemia/reperfusion FRU 30% group. **(D)** Ischemia/reperfusion TAG 30% group. 40× magnification.

The histopathological analysis revealed that the heart tissue of rats chronically fed with FRU 30% diet and subjected to I/R show a damaged structure of myofibrils and muscle fibers, surrounded by deposition of connective tissue (Figure 4C); while the morphology of heart tissue of rats fed with TAG 30% diet and subjected to I/R was comparable to heart tissue of rats fed with CTRL diet and subjected to I/R (Figures 4B,D). Figure 4A showed the heart tissue of a naive rat.

### Exposure to Fructose- or D-Tagatose-Rich Diets Differently Affects Myocardial Markers of Oxidative Stress

**FIGURE 5:**
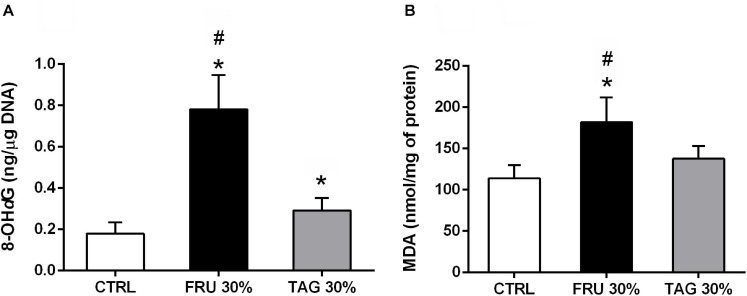
Impact of fructose- or D-tagatose-rich diets on oxidative stress markers. **(A)** 8-hydroxy-2′-deoxyguanosine (8-OH*d*G) and **(B)** malonyldialdehyde (MDA), were measured in heart of rats fed with CTRL, FRU 30% or TAG 30%. Values are represented as means ± SD of 8 (CTRL and TAG 30%) and 7 (FRU 30%) animals per group; *p < 0.05 vs. CTRL; #*p* < 0.05 vs. TAG 30%.

As shown in Figure 5, the oxidative stress markers 8-hydroxy-*d*-guanosine (8-OH*d*G, Figure 5A) and MDA (Figure 5B) recorded in the hearts exposed to I/R injury were significantly increased in rats fed with FRU 30% rich diet in comparison to animals fed with CTRL diet. In contrast, TAG 30% rich diet did not exacerbate I/R injury-induced myocardial oxidative derangements.

### Different Impact of Fructose- and D-Tagatose-Rich Diets on Systemic Cytokines Production

**FIGURE 6:**
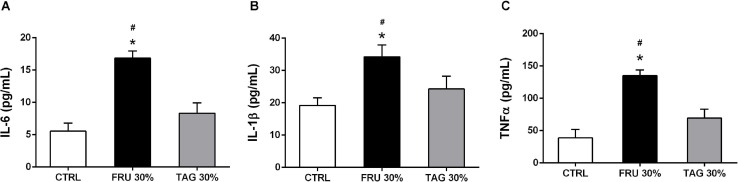
Impact of sugar feeding on systemic concentrations of cytokines. Plasmatic concentrations of interleukin-6 (IL-6) **(A)**, interleukin-1β (IL-1β) **(B),** and tumor necrosis factor-α (TNF-α) **(C)** were measured in rats fed with CTRL, FRU 30% or TAG 30%. Data are means ± SD for 14 (CTRL and TAG 30%) and 12 (FRU 30%) animals per group; *p < 0.05 vs. CTRL; #*p* < 0.05 vs. TAG 30%.

As shown in Figure 6, the systemic increase of IL-6, IL-1β, and TNF-α detected in rats exposed to I/R injury following 24 weeks of diet was significantly higher in the blood of animals fed the FRU 30%-enriched diet respect to the levels observed in the CTRL groups. On the other hand, no significant alteration in the expression of pro-inflammatory cytokines following I/R-injury were observed in plasma of animals previously fed with TAG 30%-diet, and they were not significantly different from those recorded in the control group (*p* > 0.05).

### Fructose- or D-Tagatose-Rich Diets Differently Affect Local Expression of Inflammatory Markers

**FIGURE 7:**
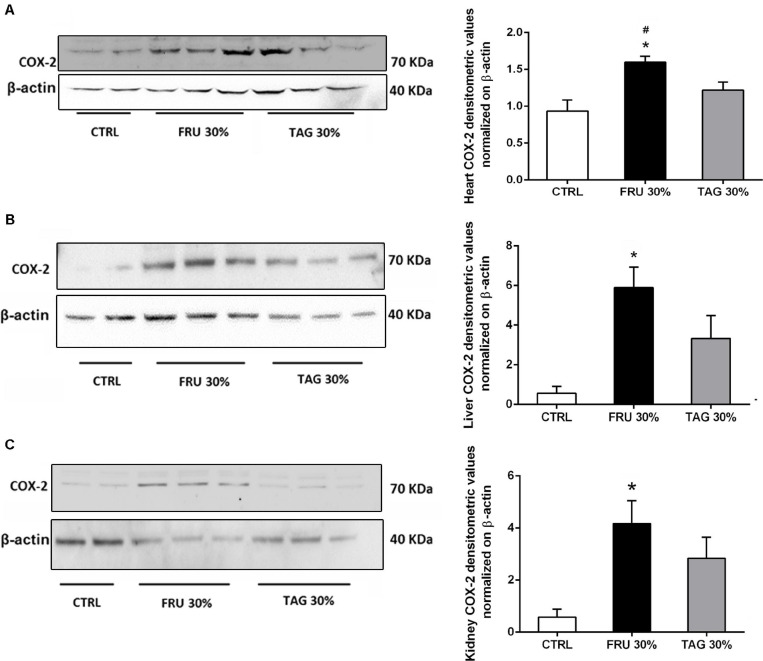
Impact of sugar feeding on myocardial, hepatic and renal expression of cyclooxygenase-2 (COX-2) enzyme isoform. Representative immunoblot and densitometric analysis of the expression of COX-2 in heart **(A)**, liver **(B)**, and kidney **(C)** of rats fed with CTRL, FRU 30% or TAG 30%. Densitometric data are reported as relative optical density (OD), corrected for the corresponding β-actin contents. Data are means ± SD for 8 (CTRL and TAG 30%) and 7 (FRU30%) samples per group; *p < 0.05 vs. CTRL; #*p* < 0.05 vs. TAG 30%.

Western blot analysis clearly demonstrates a significant increase of COX-2 expression levels in heart (Figure 7A), liver (Figure 7B), and kidney (Figure 7C) tissues of rats chronically fed with FRU 30% compared to CTRL animals. On the contrary, when compared to FRU 30%-fed rats, TAG 30% supplementation caused a less increase in COX-2 expression, with values similar to those recorded in the CTRL group when COX-2 was measured in heart tissue.

**FIGURE 8:**
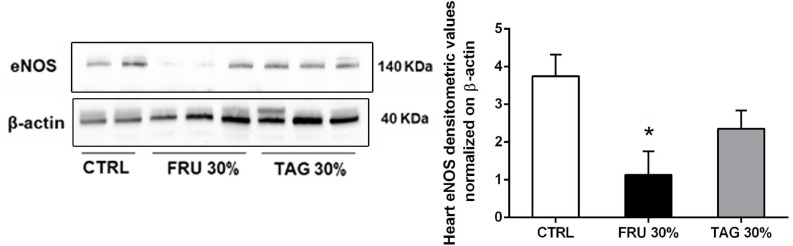
Effects of sugar feeding on cardiac expression of endothelial nitric oxide synthase (eNOS). Representative immunoblots and relative densitometric analysis of the expression of eNOS in heart samples of rats fed with CTRL, FRU 30% or TAG 30%. Densitometric data are reported as relative optical density (OD), corrected for the corresponding β-actin contents. Data are means ± SD for 8 (CTRL and TAG 30%) and 7 (FRU 30%) animals per group; *p < 0.05 vs. CTRL.

Since nitric oxide synthase (NOS) and COX systems are primarily expressed in endothelial cells and regulate the vascular function, we measured the expression of eNOS in cardiac tissue. Our results show a significant decrease of eNOS expression in heart tissue of FRU 30%-fed rats compared to CTRL group. In contrast, the chronic exposure to D-tagatose improved endothelial dysfunction by normalizing eNOS expression in the hearth tissues (Figure 8).

**FIGURE 9:**
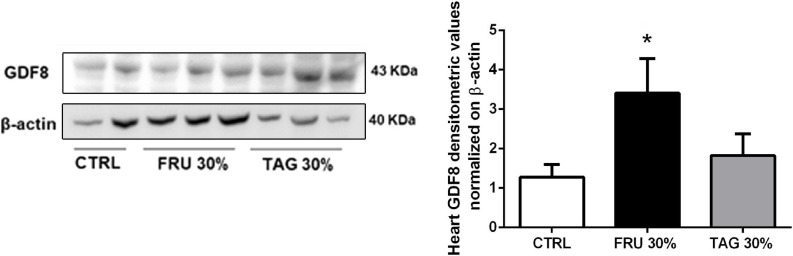
Effects of sugar feeding on expression of Growth/Differentiation Factor 8 (GDF8). Representative immunoblots and relative densitometric analysis of the expression of GDF8 in heart samples of rats fed with CTRL, FRU 30% or TAG 30%. Densitometric data are reported as relative optical density (OD), corrected for the corresponding β-actin contents. Data are means ± SD for 8 (CTRL and TAG 30%) and 7 (FRU 30%)animals per group; *p < 0.05 vs. CTRL and #*p* < 0.05 vs. TAG 30%.

It is reported that the expression of GDF8 is increased in cardiac diseases (George et al., 2010) and after myocardial infarction is up-regulated in cardiomyocytes surrounded the infarcted area (Sharma et al., 1999). Our results clearly indicate that GDF8 expression is significantly increased in heart tissue from FRU 30%-fed rats in comparison to TAG 30%-fed and CTRL animals (Figure 9).

## Discussion

Most recent data on sugar consumption from nationally representative dietary surveys across the world show that total sugar intake as a percentage of energy ranges between 13.5 and 24.6% in adults of developed countries (Newens and Walton, 2016) and clinical findings suggest that the increasing consumption of fructose may be correlated with obesity and metabolic syndrome, which are both able to induce cardiac impairment (Heidemann et al., 2008; Stanhope, 2012). Indeed, it has been hypothesized that a sugar-rich diet contributes to cardiomyocytes metabolic alterations impairing an efficient protective response and tissue recovery when heart is exposed to stress, as an ischemic event (Gonsolin et al., 2007; Pulakat et al., 2011). A study in rats has recently demonstrated that sugar-induced obesity may significantly contribute to increased propensity of the heart to malignant arrhythmias by interfering with expression and activity of myocardial electrical coupling protein, connexin-43, and protein kinase C signaling cascade (Egan Benova et al., 2019). Here we show that chronic feeding with a high fructose diet induced drastic metabolic derangements, which were paralleled by worsening of the outcomes of cardiac I/R injury, as demonstrated by drastic increases in infarct size and markers of fibrosis, inflammation and oxidative stress. Specifically, when compared to the heart of control rats, heart of rats exposed to fructose showed a robust increase in the oxidative stress markers 8-OH*d*G and MDA. The fructose ability to form weak complexes with ferric iron, as previously demonstrated (Paterna et al., 1998), may significantly contribute to alter the intracellular production of oxygen free radicals, therefore resulting in impairment of oxidative stress markers. Similarly, heart of fructose-fed rats showed over-expression of the pro-inflammatory enzyme COX-2 associated with decreased expression of eNOS isoform, suggestive of reduced local production of NO and, thus, endothelial dysfunction. COX-2 was maximally expressed not only in the heart but also in the kidney and liver of rats chronically fed with fructose, thus indicating a multi-organ inflammatory response, confirmed by the robust increased concentrations of pro-inflammatory cytokines in the blood of fructose-fed rats. Interestingly, in hearts of fructose-fed rats, we also documented a statistically significant increase in GDF8, which has been reported to contribute to accelerate the progression of fibrosis and late dysfunction in infarcted and remodeling hearts by promoting the proliferation of fibroblasts and expression of extracellular matrix proteins (Lim et al., 2018). Overall, these findings suggest that the causal link between overconsumption of fructose and increased myocardial susceptibility to I/R injury is due, at least in part, to an excessive inflammatory response associated with impairment in the oxidant/antioxidant balance. Our data are in keeping with previous findings showing that excessive intake of fructose increases the cellular production of ROS (Jaiswal et al., 2015). The increased levels of ROS in cardiac tissue following I/R has been reported to contribute to reduced expression of cardiac NOS (de Castro et al., 2015), thus impairing the ability of coronary arteries to dilate, and thus, resulting in increased myocardial susceptibility to I/R injury (Prakash et al., 2011). Besides, a decreased NOS activity leads to an increased expression of COX-2 and consequent production of pro-inflammatory mediators (Meza et al., 2019).

We previously showed that chronic fructose feeding in mice resulted in massive increase in markers of oxidative stress, radical-induced DNA damage and inflammatory response in the heart and these effects were not recorded when animals were fed with D-tagatose diet (Collotta et al., 2018). Here we further extended our previous findings in a different animal specie to the comparative impact of the two sugar isomers on cardiovascular risk associated with metabolic derangements. Specifically, we demonstrated that D-tagatose does not exacerbate the I/R-induced oxidative stress and related inflammatory response, which were maximal in hearts of rats exposed to I/R after chronic fructose feeding. As elevated expression of COX-2 in cardiomyocytes has been associated with heart failure (Abbate et al., 2004), the lack of significant effects on COX-2 expression corroborates the evidence of reduced cardiac toxicity, even after I/R injury, in D-tagatose diet when compared to fructose feeding. The significant difference in expression levels of hydroxyproline, whose measurements accurately reflect the amount of collagen in the tissue, and GDF8, a reliable marker of clinical severity after acute myocardial infarction (Meloux et al., 2019), which were both maximally upregulated in the I/R heart of fructose-fed rats and only slightly overexpressed in the presence of D-tagatose, is a further evidence of the limited toxic impact of D-tagatose diet on cardiac damage after I/R when compared to a fructose rich diet.

As far as we know, this paper represents the first observation regarding different myocardial susceptibility following I/R injury after chronic intake of two sugar isomers, fructose or D-tagatose. Our data demonstrate that altering the content of a single nutrient in a diet is enough to reduce the risk of diet-induced metabolic derangements and related exacerbation of cardiovascular injury. In contrast, a diet containing an equivalent amount of D-tagatose had only slight effect on body weight, systemic lipid and glucose profiles, as well as on insulin level and insulin resistance index. These findings are in agreement with previous findings showing that D-tagatose, as a carbohydrate source, did not promote obesity and hyperglycemia and these effects were associated with lower risk of hypercholesterolemia and atherosclerosis, in comparison to sucrose, when tested in low-density lipoprotein receptor deficient (LDLr^–/–^) mice (Police et al., 2009). Overall, the results here reported strengthen the evidence on health promoting effects of D-tagatose. In fact, D-tagatose shows several beneficial properties besides its sweetness, such as low glycemic index, reduced energy value, prebiotic and antioxidant effects (Roy et al., 2018). Thus, thanks to is capability to prevent the lifestyle related diseases, it can be used as a low caloric bulk sweetener in a wide variety foods, health products, beverages, and dietary supplements.

The experimental model here proposed allows us to compare the impact of chronic exposure to the two fructose isomers on the heart injury evoked by acute I/R challenge in a strictly controlled environment, avoiding confounding factors. However, we are aware of some limitations of the present study, including the high content of dietary sugars, which are beyond average human consumption, the lack of investigation on potential synergistic effects of the fructose isomers with other relevant dietary components, the impossibility to dissect causal relationships between changes in cellular activation of redox and inflammatory cascades evoked by sugar exposure and the specific heart cell types mainly affected. We also did not consider the sugars’ impact on gut microbiota and integrity, being D-tagatose partially fermented in the large intestine and used by gut microbiota as substrate (Liang et al., 2019). Differences between the two fructose isomers in terms of digestibility might contribute to explain, at least in part, their different toxicological profiles. The acute myocardial infarction model we used is generally recognized to mimic human ischemic heart disease and it serves as an important tool to elucidate the molecular signaling mechanisms and to study the consequence of a myocardial infarction on cardiac pathophysiological function (Masini et al., 2002; Bianchi et al., 2005; Collino et al., 2012). Nevertheless, the beneficial impact of D-tagatose feeding here documented has to be confirmed also in suitable models of chronic cardiovascular injury to strengthen the perspective of the beneficial effects of D-tagatose as sugar alternative in cardiometabolic settings. Thus, future *ad hoc* studies, with implemented technologies, are required to clarify these issues as well as to better elucidate its safety profile.

## Conclusion

In conclusion, here, we demonstrated for the first time that chronic exposure to D-tagatose exerts less harmful effects on myocardial susceptibility to I/R injury, when compared to fructose, thus strengthening the rationale for the use of D-tagatose as safer paradigm of sweeteners with limited toxicological impact not only on metabolic disorders but also on the related cardiovascular risks. Overconsumption of sugar containing food and beverages contributes to the development of obesity and diabetes epidemic. In 2016, the World Health Organization reported in Western countries, such as United States of America, 64.5% population was overweight (Jacques et al., 2019) and over the last 10 years the prevalence of obesity across the European continent has in general been rising (Blundell et al., 2017). Furthermore, the fructose-derivative sugar could have several advantages over the most widely used simple carbohydrates, for reducing the risk of CVD in western populations, including those with metabolic derangements.

## Data Availability Statement

The raw data supporting the conclusions of this article will be made available by the authors, without undue reservation.

## Ethics Statement

The animal study was reviewed and approved by the Animal Ethical and Care Committee of the University of Florence (Florence, Italy) and the Italian Health Ministry (Authorization n 1189/2016-PR).

## Author Contributions

EM and MC: conceptualization and funding acquisition. MD, SS, LL, EM, and MC: methodology. GP: software and formal analysis. MD, SS, PF, AL, and LL: investigation. EM, MC, and GP: data curation. MC and SS: writing original draft preparation. LL, DC, and EM: writing review and editing. MC: supervision. EM: project administration. All authors have read and agreed to the published version of the manuscript.

## Conflict of Interest

The authors declare that the research was conducted in the absence of any commercial or financial relationships that could be construed as a potential conflict of interest.
