# Supplementary material for: D-Tagatose Feeding Reduces the Risk of Sugar-Induced Exacerbation of Myocardial I/R Injury When Compared to Its Isomer Fructose
Source: Front Mol Biosci. 2021 Apr 13;8:650962. doi: 10.3389/fmolb.2021.650962 (PMC8076855; doi:10.3389/fmolb.2021.650962)

COX-2 and β actin in heart tissue

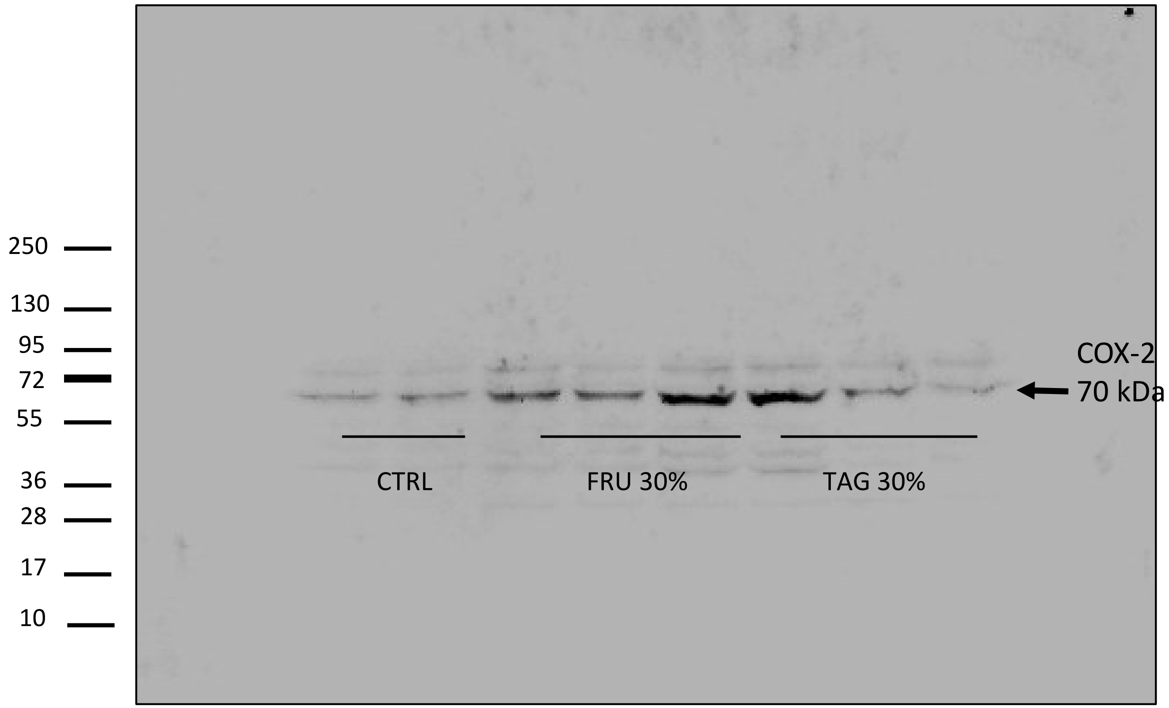

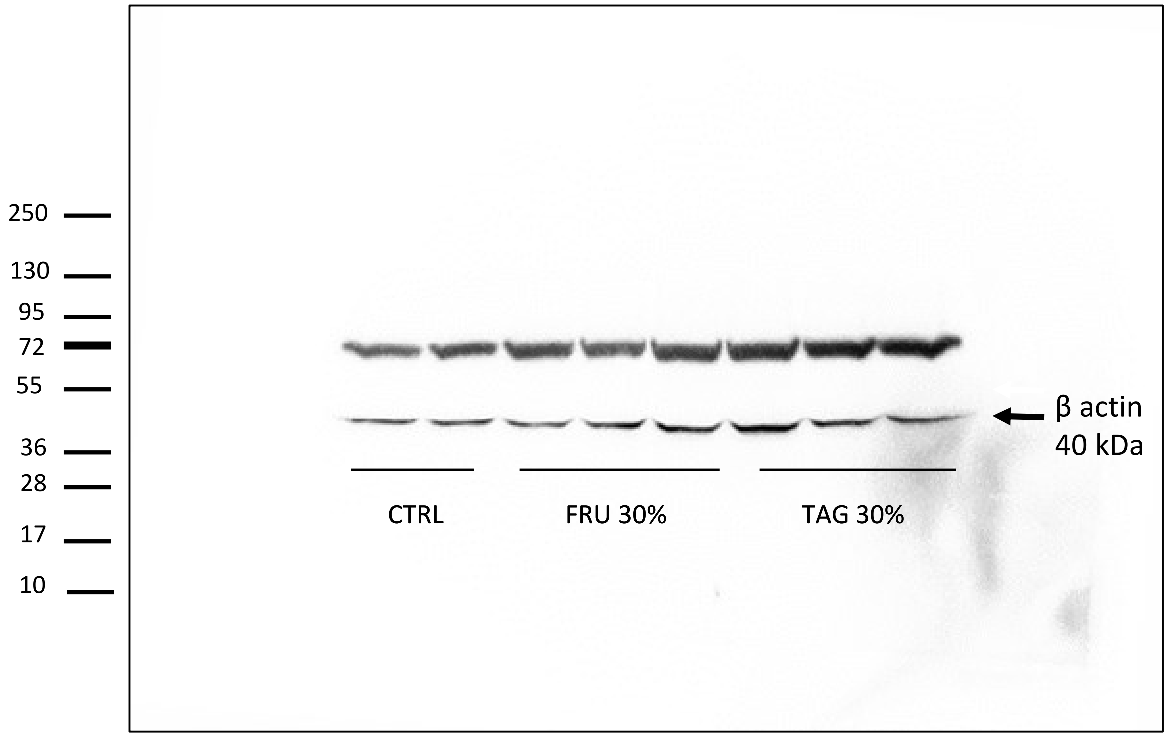

COX-2 and β actin in liver tissue

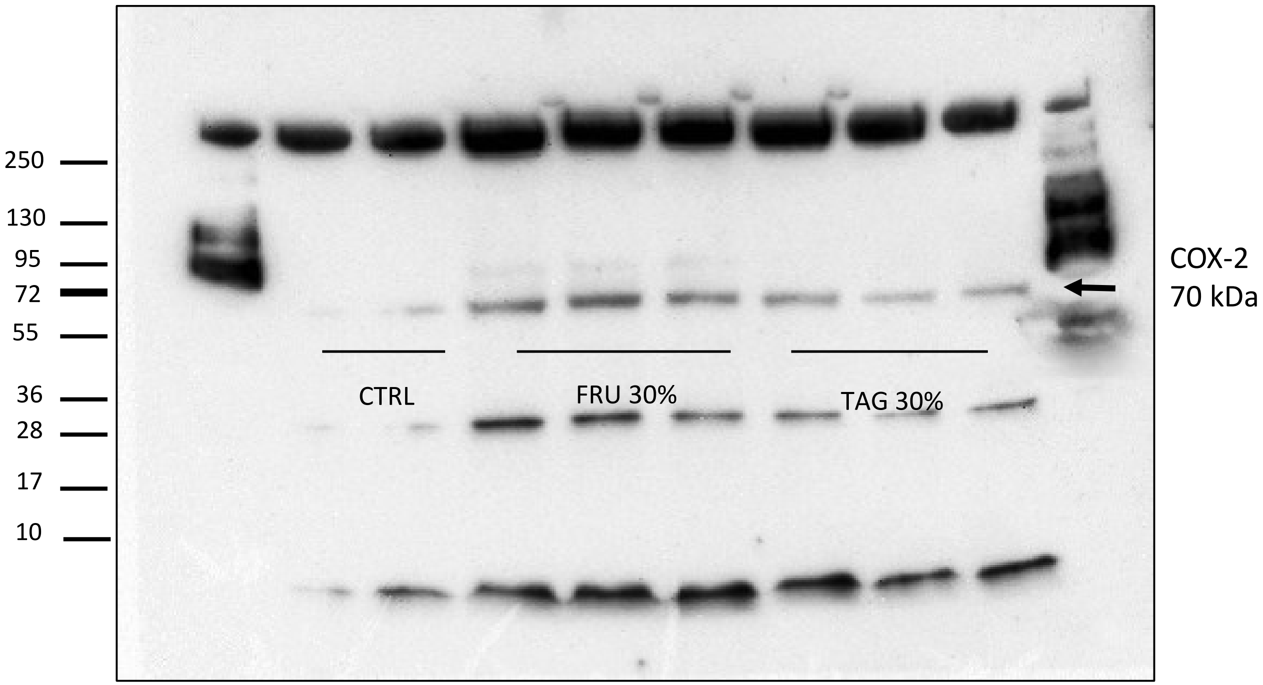

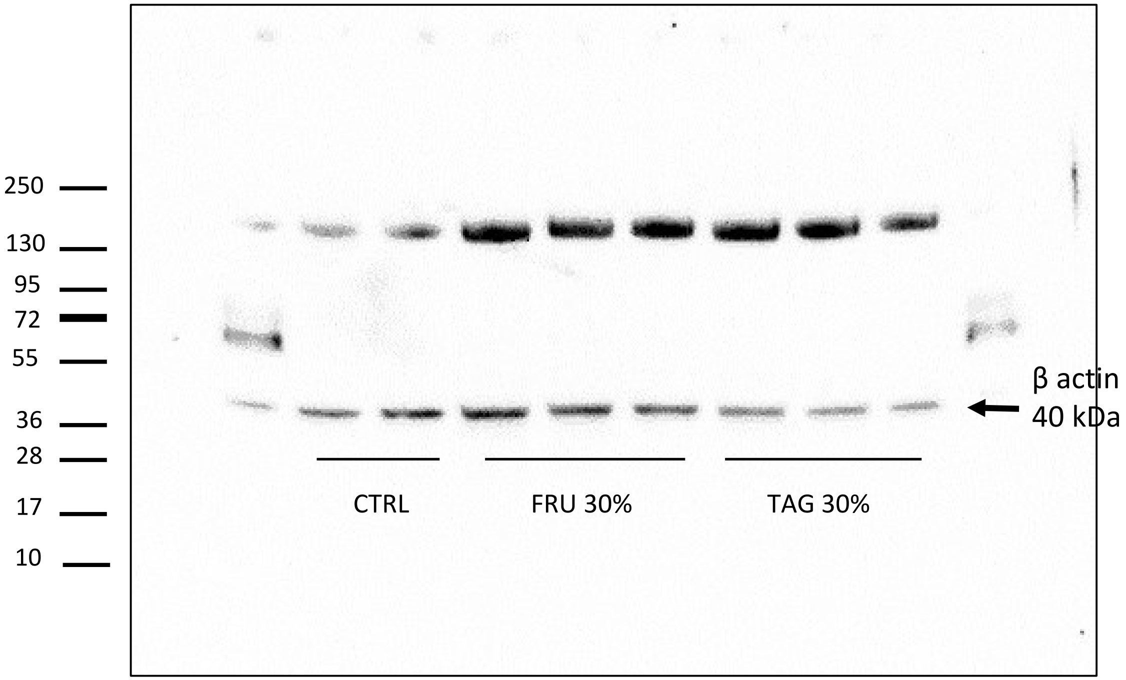

COX-2 and β actin in kidney tissue

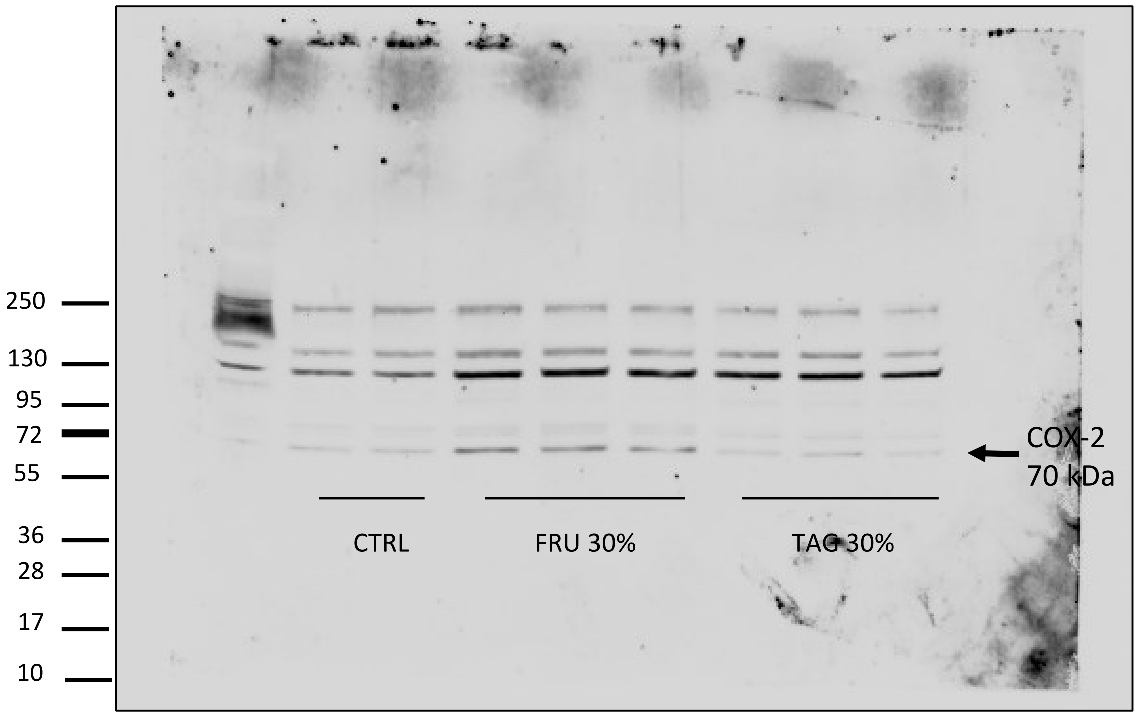

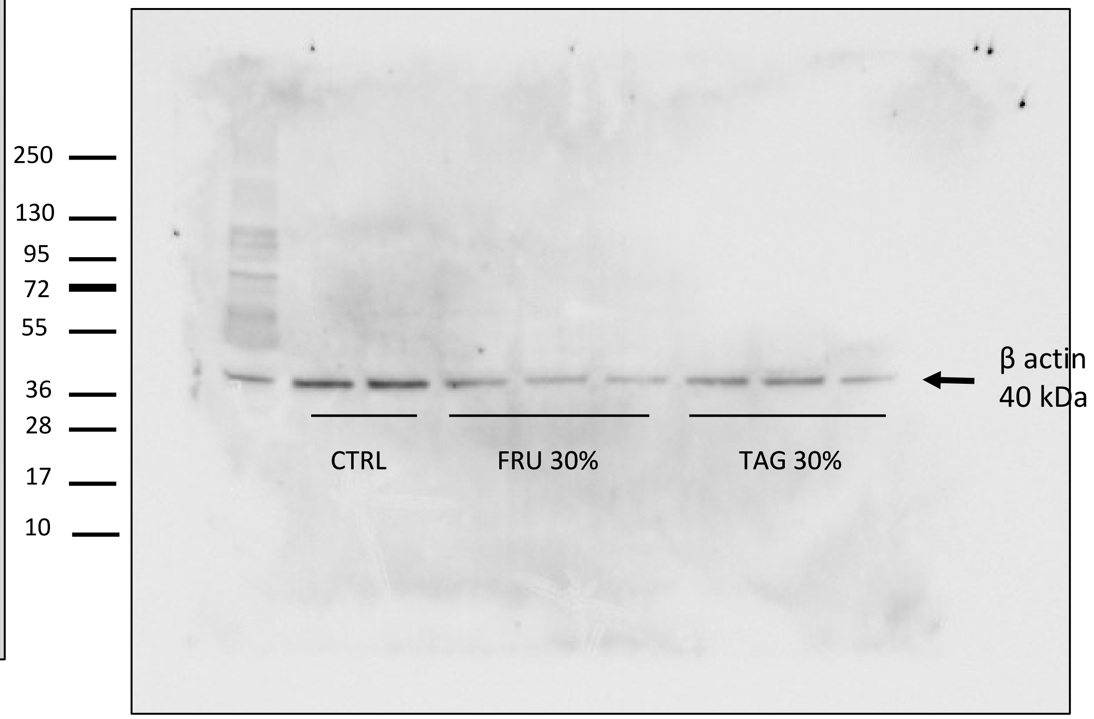

eNOS and β actin in heart tissue

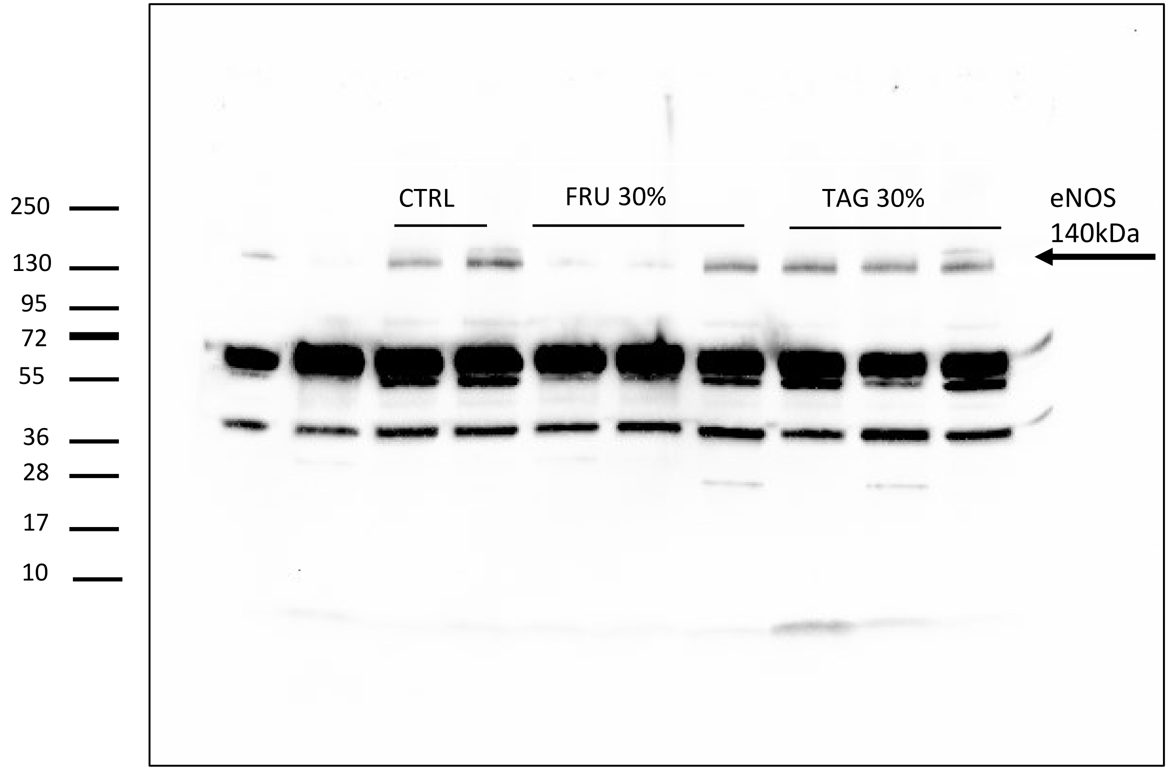

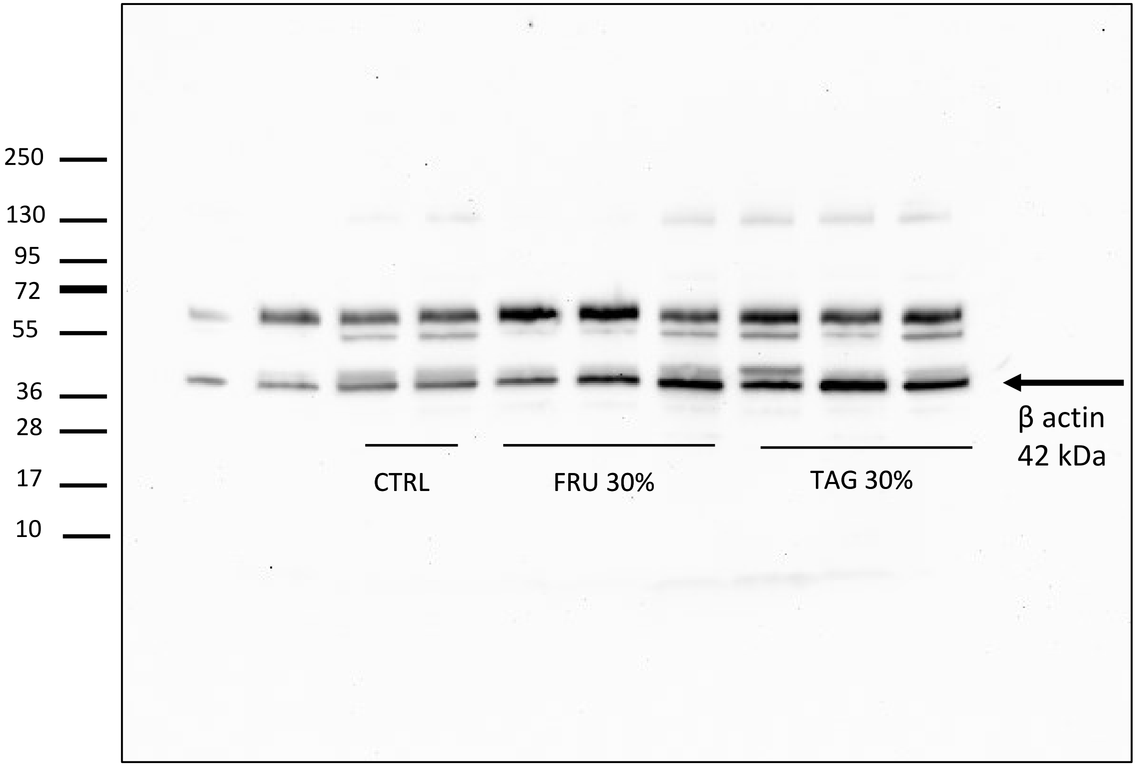

GDF8 and β actin in heart tissue

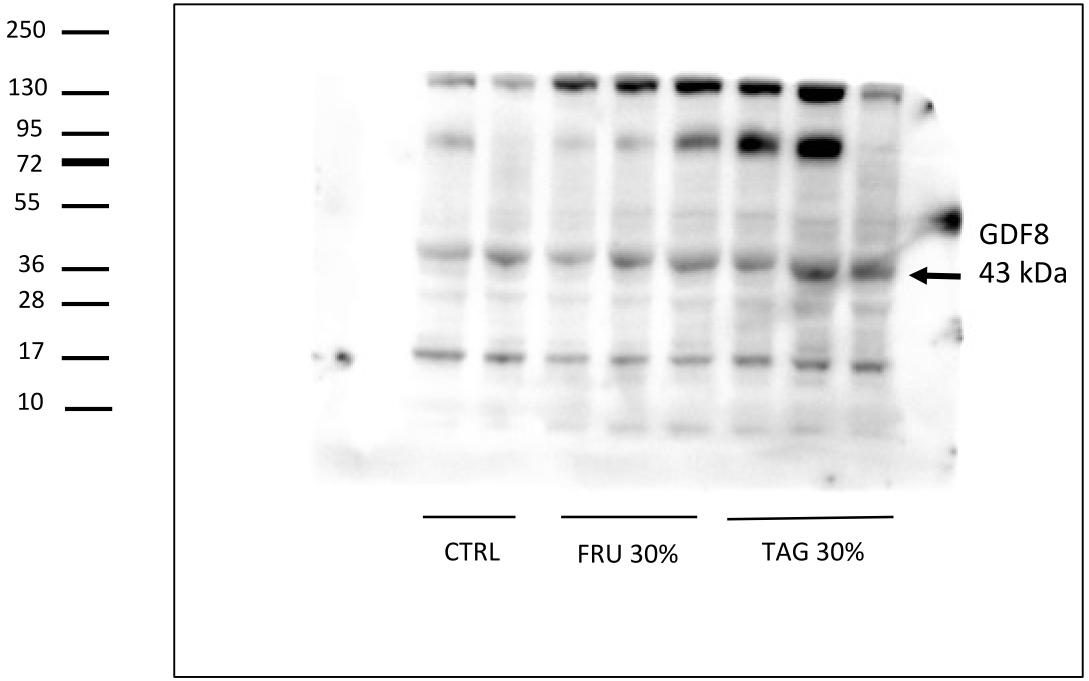

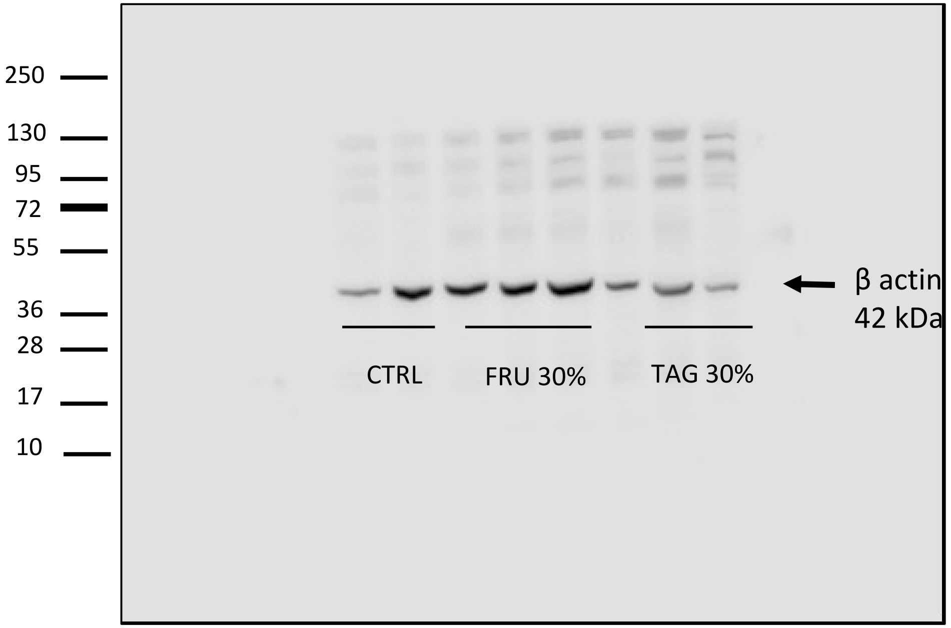

Supplement: Supplementary file 1 [file Data_Sheet_1.PDF]
